# Activating frataxin expression by repeat-targeted nucleic acids

**DOI:** 10.1038/ncomms10606

**Published:** 2016-02-04

**Authors:** Liande Li, Masayuki Matsui, David R. Corey

**Affiliations:** 1 Departments of Pharmacology and Biochemistry, UT Southwestern Medical Center at Dallas, 6001 Forest Park Road, Dallas, Texas 75390-9041, USA

## Abstract

Friedreich's ataxia is an incurable genetic disorder caused by a mutant expansion of the trinucleotide GAA within an intronic *FXN* RNA. This expansion leads to reduced expression of frataxin (FXN) protein and evidence suggests that transcriptional repression is caused by an R-loop that forms between the expanded repeat RNA and complementary genomic DNA. Synthetic agents that increase levels of FXN protein might alleviate the disease. We demonstrate that introducing anti-GAA duplex RNAs or single-stranded locked nucleic acids into patient-derived cells increases FXN protein expression to levels similar to analogous wild-type cells. Our data are significant because synthetic nucleic acids that target GAA repeats can be lead compounds for restoring curative FXN levels. More broadly, our results demonstrate that interfering with R-loop formation can trigger gene activation and reveal a new strategy for upregulating gene expression.

## Abstract (web summary)

Expansion of the trinucleotide GAA within an intronic FXN RNA can cause Friedreich's Ataxia (FRDA), an incurable genetic disorder. Here, the authors show that anti-GAA duplex RNAs or single-stranded locked nucleic acids increases FXN protein expression in patient-derived cells to levels similar to wild-type cells.

## Graphical abstract

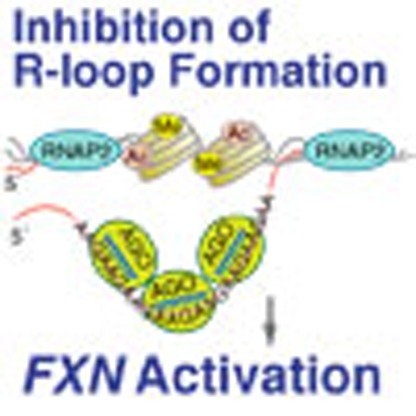

Friedreich's ataxia (FRDA) is an incurable genetic disorder caused by reduced expression of the mitochondrial protein frataxin (FXN)^1^,^2^. FRDA patients have an expanded GAA-repeat region within the first intron of the *FXN* gene (Fig. 1a). Reduced expression of *FXN* is associated with changes in chromatin modification including increased H3K9 methylation and decreased H3K9 acetylation^3^,^4^,^5^,^6^.

Unlike neurological diseases such as Huntington Disease that are caused by the expression of mutant protein, patients with FRDA express normal FXN protein but do so at a reduced level. The progression of the disease would likely be slowed if FXN levels could be enhanced and agents that increase expression of *FXN* are a promising approach to therapy^7^,^8^,^9^,^10^.

Potential approaches for increasing *FXN* expression include the use of histone deacetylase inhibitors to reverse the epigenetic changes that contribute to decreased expression^6^,^10^,^11^,^12^,^13^. A screen of existing drugs identified the topical anaesthetic dyclonine as an activator of *FXN* expression, possibly by inducing expression of the transcription factor Nrf2 (ref. 14). While promising, these strategies rely on nonspecific gene activation, a potential disadvantage that may slow progress towards clinical use.

Evidence suggests that the expanded GAA tract within the nascent *FXN* transcript binds to genomic DNA to form an R-loop^15^,^16^,^17^,^18^,^19^. This R-loop may then interfere with transcription and reduce *FXN* expression (Fig. 1b), possibly by inducing silencing chromatin modifications that impeded transcriptional elongation^20^,^21^. Synthetic nucleic acids complementary to the GAA repeat might bind to the expanded RNA, block R-loop formation and remove the trigger for transcriptional silencing. We set out to test this hypothesis using repeat-targeted duplex RNAs and single-stranded locked nucleic acid (LNA) oligonucleotides.

Our laboratory and others have developed strategies for using antisense oligonucleotide and duplex RNAs to recognize the expanded CAG trinucleotide repeats including mutant huntingtin (Huntington disease), ataxin-3 (Machado Joseph disease) and atrophin-1 (Dentatorubral-pallidoluysian atrophy)^22^,^23^. There are major differences, however, between the CAG-containing messenger RNAs (mRNAs) and *FXN* that affect experimental design and potential mechanisms of action. In contrast to anti-CAG duplexes, nucleic acids that target the expanded *FXN* repeat must: (1) target a GAA repeat; (2) activate rather than inhibit gene expression; (3) target a sequence within an intron rather than an exon and (4) act in the nucleus on intronic RNA rather than the cytoplasm on mature mRNA.

Duplex RNAs can act in the nucleus to enhance transcription of specific target genes or alter splicing^24^,^25^. The mechanism of action involves recognition of an RNA transcript associated with the genomic loci and recruitment of argonaute 2 (Ago2) and other RNAi factors. Both Ago2 and other RNAi factors are present in cell nuclei^26^ and appear to be critical elements of the cellular machinery driving recognition.

In this study we test the hypothesis that repeat-targeted RNAs can activate expression of the *FXN* gene. We show that duplex RNAs and single-stranded LNAs targeting the GAA-repeat activate expression of *FXN* RNA and protein. These data suggest that anti-GAA nucleic acids can be lead compounds for the development of agents for restoring therapeutic levels of FXN protein.

## Results

### Design of anti-GAA duplex RNAs

**Figure 1:**
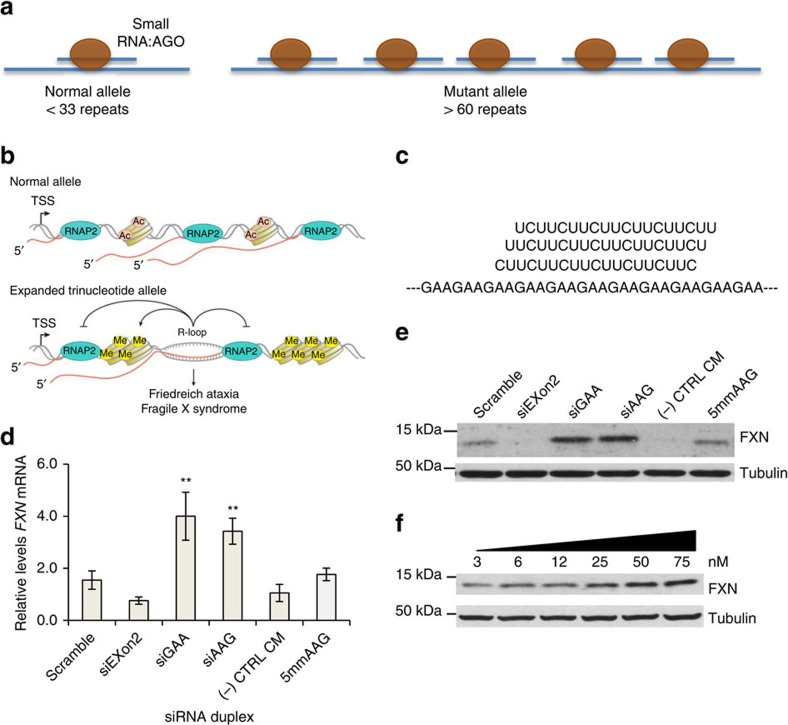
RNA-mediated activation of *FXN* expression. (**a**) Schematic of repeat expansion within intronic *FXN* mRNA and binding of AGO:RNA complexes. The longer mutant repeat is predicted to bind more AGO:RNA complexes than the shorter wild-type repeat. (**b**) Schematic of R-loop formation at *FXN* locus and potential to influence histone modification and gene expression (Adapted from Groh *et al.*^19^). (**c**) Complementarity of guide strand RNAs (25 nM) to *FXN* RNA. (**d**,**e**) Effect of anti-GAA duplex RNAs on **d**, *FXN* mRNA (*n*=3) and **e**, protein expression. (**f**) A dose-response profile of upregulation of FXN protein expression by siGAA. FRDA patient-derived fibroblast cells (GM03816) were used. siExon2 is a duplex siRNA that targets *FXN* exon and is expected to decrease *FXN* expression. CM is a negative control RNA that is not complementary to *FXN* RNA. RNA scramble is a duplex RNA in which the sequences of siGAA and siAAG are mixed to preserve nucleotide composition by alter their order. si5mmAAG is similar to siAAG but has five mismatches relative to the repeat region within FXN mRNA target. Cells were collected at day 3 for RNA extraction and day 4 for protein extraction. Error bars:±STDEV. **P<0.01, by Student *t*-test.

We designed duplex RNAs complementary to the GAA repeat within *FXN* pre-mRNA (Fig. 1c, Supplementary Table 1). The anti-GAA RNAs or control duplexes were transfected into GM03816 patient-derived fibroblast cells using cationic lipid. Three different anti-GAA duplexes each possessed a different register (siAAG, siAGA and siGAA) relative to the repeat target. The duplex RNAs were either fully complementary to the mutant *FXN* repeat or contained central mismatches. Duplex RNAs that contain central mismatches can bind to target RNAs but cannot engage the cleavage function of Ago2 (ref. 27).

### Anti-GAA RNAs upregulate FXN expression

We introduced duplex RNAs into patient-derived GM03816 cells and observed increased expression of FXN mRNA and protein (Fig. 1d–f, Supplementary Fig. 1). Upregulation of *FXN* mRNA levels was three to fourfold while protein levels were increased four to sixfold. Half maximal activation was achieved after addition 10–15 nM RNA (Fig. 1f). This potency is typical of that observed for other activating synthetic RNAs^25^. Activation continued to be observed 15 days after addition of duplex RNA (Supplementary Fig. 2). Addition of anti-GAA duplex RNAs did not upregulate FXN protein expression in a fibroblast cell line, GM02153, with wild-type levels of FXN (Supplementary Fig. 3).

Negative control duplex RNAs containing scrambled sequences, five mismatched bases or that had no complementarity did not activate *FXN* expression. One source of off-target effects by oligonucleotides is induction of interferon responsive genes, but no activation of interferon responsive genes was observed (Supplementary Fig. 4). We also measured expression of *NEXN-AS1* and *PRDM10*, genes containing 32 or 10 GAA repeats respectively (Supplementary Fig. 5). Expression levels of the GAA-repeat genes were unchanged.

**Figure 2:**
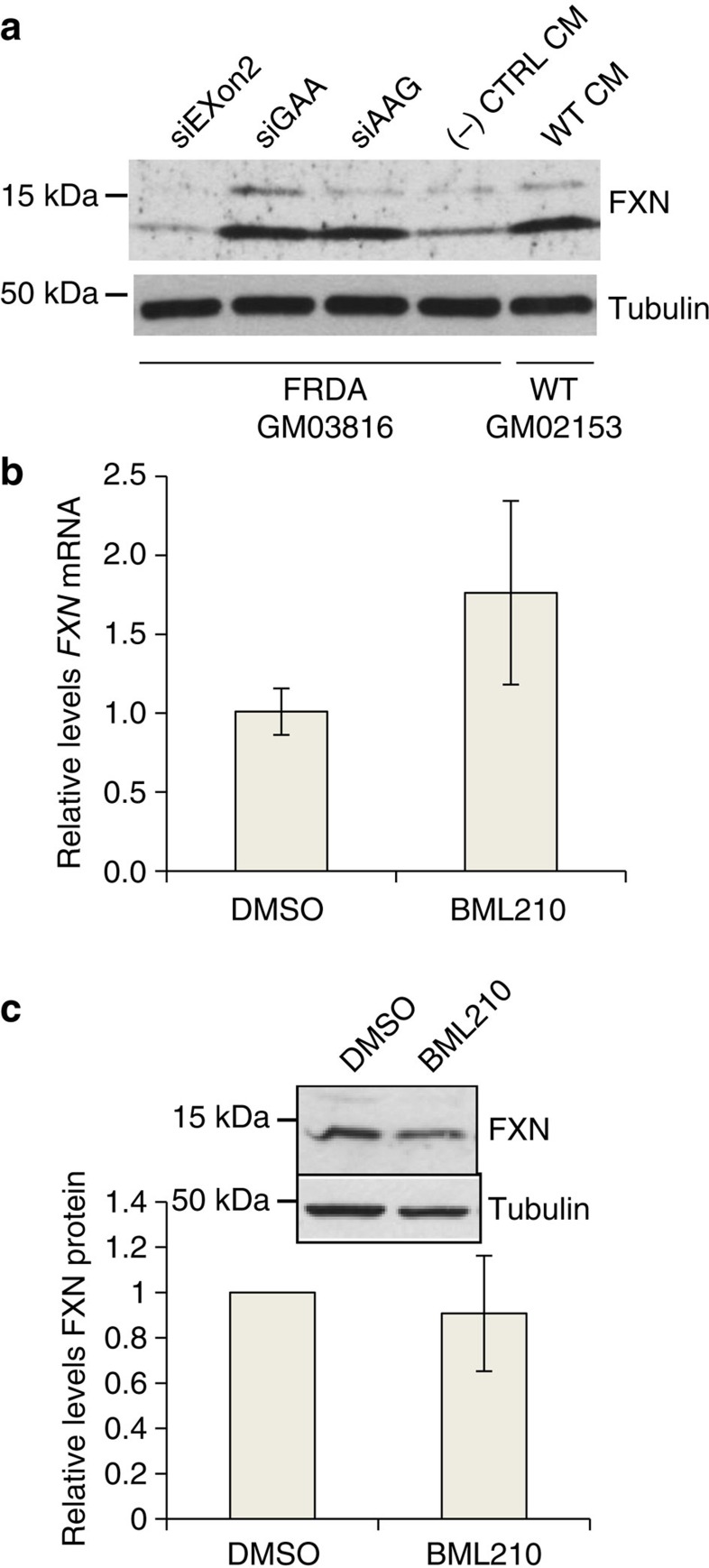
Comparison of RNA-mediated activation of *FXN* expression in patient cells to *FXN* expression in normal cells and *FXN* activation by a histone deacetylase inhibitor. (**a**) Activation of FXN protein expression in FRDA cells (GM03816) and wild-type fibroblast cells (GM02153). (**b**,**c**) Effect of HDAC inhibitor BML210 (5 μM) treatment on expression of **b** *FXN* mRNA (*n*=3) and (**c**) protein (inset, western analysis, *n*=4) expression in FRDA patient fibroblast cells (GM03816). All data are presented as mean±STDEV.

The levels of FXN protein on addition of activating duplex RNAs were similar to the wild-type levels in GM02153 cells (Fig. 2a, Supplementary Fig. 6), suggesting that RNA-mediated upregulation can induce expression of physiologically relevant amounts of the protein. For comparison we examined levels of *FXN* expression in patient-derived GM03816 cells that had been treated with BML210, a small molecule known to induce histone modifications and has been reported to cause activation of *FXN*^28^. Averaged quadruplicate experiments did not reveal a significant increase in *FXN* mRNA or protein levels on treatment of GM03816 cells with BML210 (Fig. 2b,c).

### Duplex RNAs recruit Ago2 to mutant intronic GAA repeat

Our activating RNAs are double-stranded and identical in design to siRNAs typically used for RNA silencing. The duplex RNAs may, therefore, be acting through the RNAi pathway in cell nuclei by binding to the expanded repeat and recruiting RNAi factors. One factor, Ago2 is the central protein component of the cellular RNAi machinery^29^. Ago2 binds the guide strand of duplex RNA and participates in efficient recognition of mRNA. We had previously observed that transfection of RNAs that activate progesterone receptor or cyclooxygenase-2 expression lead to binding of the Ago2 to the target transcript overlapping the gene promoters^25^.

**Figure 3:**
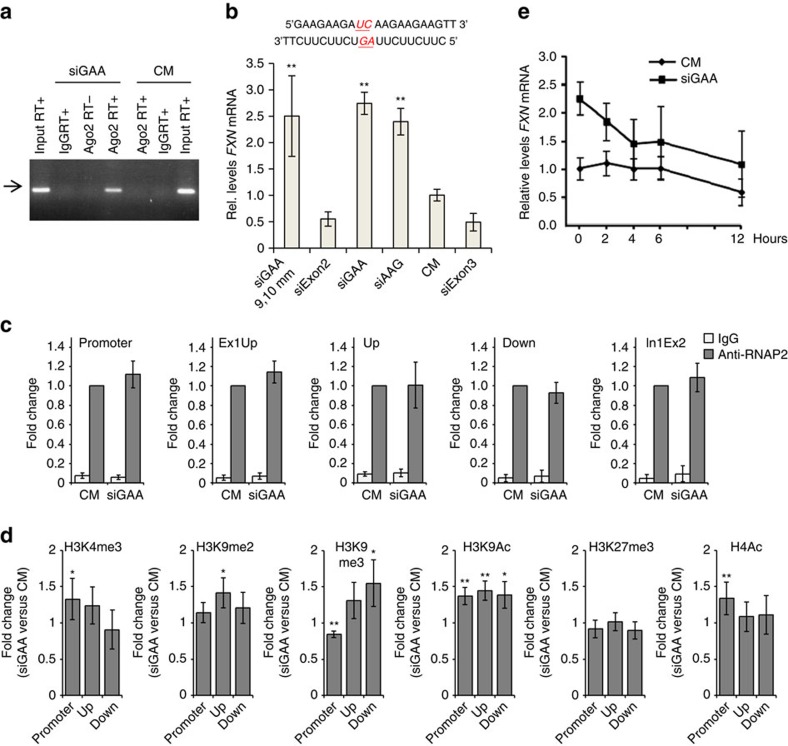
Mechanism of *FXN* activation by repeat-targeted duplex RNAs. (**a**) RIP examining the association of Ago2 with *FXN* pre-mRNA after treatment with 50 nM duplex RNA and analysis by real-time PCR. An arrow marks the PCR product of *FXN* pre-mRNA, which was confirmed by sequencing (Supplementary Fig. 7) (**b**) Anti-GAA duplex RNA with central mismatches (siGAA 9,10 mm with mismatches on both strands; 25 nM) activates *FXN* expression at a level similar to the analogous fully complementary duplex RNA (*n*=3). siExon3 is a positive control for transfection efficiency targeting exon 3 of *FXN*. (**c**) ChIP for RNAP2 using four different primer sets (*n*=4). (**d**) ChIP for transcription-associated histone modification markers H3K4me3, H3K9me2, H3K9me3, H3K9Ac, H3K27me3 and H4Ac (*n*=4–8). (**e**) *FXN* mRNA stability assay. Cells were transfected with duplex RNAs siGAA or CM at 25 nM (*n*=3). Actinomycin D (5 μg ml^−1^) was added with fresh media 3 days after transfection and cells were collected at the indicated time points. HPRT expression was measured for normalization. All experiments were performed in GM03816 patient-derived cells. All data are presented as mean±STDEV. *P<0.05, **P<0.01, by Student *t*-test.

We examined recognition of the repeat-containing intronic transcript and RNA:Ago2 complex using RNA immunoprecipitation (RIP) with an anti-Ago2 antibody. We transfected cells with anti-GAA RNA, isolated RNA from cell nuclei, and purified Ago-bound RNA. Analysis of immunoprecipitated RNA by reverse transcriptase PCR revealed binding of Ago2 to *FXN* pre-mRNA repeat when synthetic RNA complementary to the GAA repeat was added to cells (Fig. 3a, Supplementary Fig. 7). These data suggest that the first step in the mechanism of activation is RNA-mediated recruitment of Ago2 to the target transcript.

### No requirement for Ago2-mediated cleavage of FXN RNA

Ago2 will cleave target mRNA if the strands have perfect complementarity at key central bases^28^. If central mismatches are introduced, however, Ago2 will not cleave the target strands but will retain the ability to bind^27^. This is a useful feature for investigating mechanism because it allows the potential for strand cleavage to be evaluated for its contribution to RNA-mediated control of gene expression. To determine whether cleavage of the transcript was essential for activating *FXN* expression, we evaluated anti-GAA RNA (siGAA9,10 mm) that contained mismatched bases in its central region relative to the repeat region at positions 9 and 10 (Fig. 3b). This mismatch-containing RNA-activated *FXN* expression as well as fully complementary duplexes siGAA or siAAG, demonstrating that cleavage of the target transcript is not a necessary feature of the mechanism of action. Our finding demonstrates that binding of the RNA-Ago2 complex is sufficient to trigger activation and is consistent with a mechanism of action that involves blocking association with *FXN* genomic DNA.

### Activating histone modifications

We performed chromatin immunoprecipitation (ChIP) for RNA polymerase 2 (RNAP2) to investigate whether the association of RNAP2 with the *FXN* locus changes on anti-GAA duplex RNA treatment. We observed no enhanced recruitment of RNAP2 at the *FXN* gene promoter and four regions downstream (Fig. 3c, Supplementary Table 3). This finding is consistent with a previous report that there was no difference in levels of phosphorylated RNAP2 between the wild-type and FRDA patient-derived lymphocytes^30^.

We also performed ChIP to investigate how activating duplex RNA affect levels of histone modifications at the *FXN* gene locus. Changes of histone acetylation in the promoter and regions upstream and downstream of the GAA repeats in the first intron of *FXN* gene have been investigated previously^8^,^27^. The studies revealed decreased levels of acetylated histone H3 and H4 (H3K9Ac, H3K14Ac, H4K5Ac, H4K8Ac, H4K12Ac and H4K16Ac) in the FRDA lymphoid cell line (GM15850)^27^ or human tissues^8^. Treatment with HDAC inhibitor BML210 increased acetylation of histone H3 and H4 leading to upregulation of *FXN* mRNA and protein in the lymphoid cells or primary lymphocytes^27^.

We measured levels of H3K9Ac and H4Ac in the promoter and regions upstream and downstream of the GAA repeats on control or anti-GAA duplex RNA treatment (Fig. 3d). Our duplex RNA targeting the GAA repeats increased H3K9Ac levels by ∼1.4-fold relative to the control duplex RNA in the promoter and upstream/downstream regions of the GAA repeats, while H4Ac levels increased by ∼1.3-fold only in the promoter region. These results suggest that our activating duplex RNA can reverse histone acetylation associated with the repeat expansion and lead to activation of *FXN* gene.

We also investigated levels of H3K4me3, H3K9me2, H3K9me3 and H3K27me3 (Fig. 3d). Previous research reported increased levels of di- and tri-methylated histone H3 correlated with DNA hypermethylation at the upstream region^7^,^8^ and hypomethylation at the downstream region^8^ of the repeats in lymphoblasts, brain or heart tissues derived from FRDA affected individuals. On transfection of activating anti-GAA duplex RNA into FRDA fibroblast cells, we observed increase of H3K9me2 or H3K9me3 levels (up to 1.5-fold) at the upstream and/or downstream regions of the repeats, showing that upregulation of *FXN* gene by duplex RNA couples with change of histone methylation. These results suggest that our duplex RNA change status of histone methylation as well as histone acetylation at the *FXN* gene locus and relieve heterochromatin-mediated repression of *FXN* gene.

To determine whether enhanced protein stability might be involved in gene activation we examined FXN mRNA stability upon treatment with anti-GAA duplex RNA. Actinomycin D interferes with RNA synthesis and allows the persistence of RNA to be monitored over time. We observed that treatment with anti-GAA duplex RNA did not lead to stabilization of *FXN* RNA relative to treatment with control duplex (Fig. 3e).

These protein stability data, together with our data showing unchanged recruitment of RNAP2, suggests that RNA activation by anti-GAA duplex RNAs occurs at the level of RNA synthesis but not at the level of polymerase binding. Increased expression that is not dependent on increased recruitment of RNAP2 is consistent with previous observations of the mechanism of impaired elongation in FRDA patient-derived cells^20^,^21^,^30^. Our observation of RNA-induced histone modifications is also consistent with relief of heterochromatin formation, suggesting that transcript elongation is being enhanced.

R-loop formation is measured by immunodetection of the RNA–DNA hybrid at a site of interest. Using this method, DNA immunoprecipitation (DIP), Gromak and colleagues have shown that R-loops promote gene silencing in FRDA patient-derived cells.^18^ We observed that addition of anti-AAG RNAs reversed R-loop formation (Supplementary Fig. 8). The finding that reversed R-loop formation correlates with elevated FXN protein expression is consistent with predictions by Gromak^18^.

### Single-stranded oligonucleotides activate FXN expression

Duplex RNAs have the potential to target either sense or antisense transcripts at the *FXN* locus. To further define the molecular target and mechanism we tested anti-GAA LNA oligomers for their ability to affect *FXN* expression (Supplementary Table 2). Single-stranded LNAs were designed to be complementary to either *FXN* pre-mRNA or a complementary antisense transcript, but not both, allowing the molecular target to be more accurately assessed.

**Figure 4:**
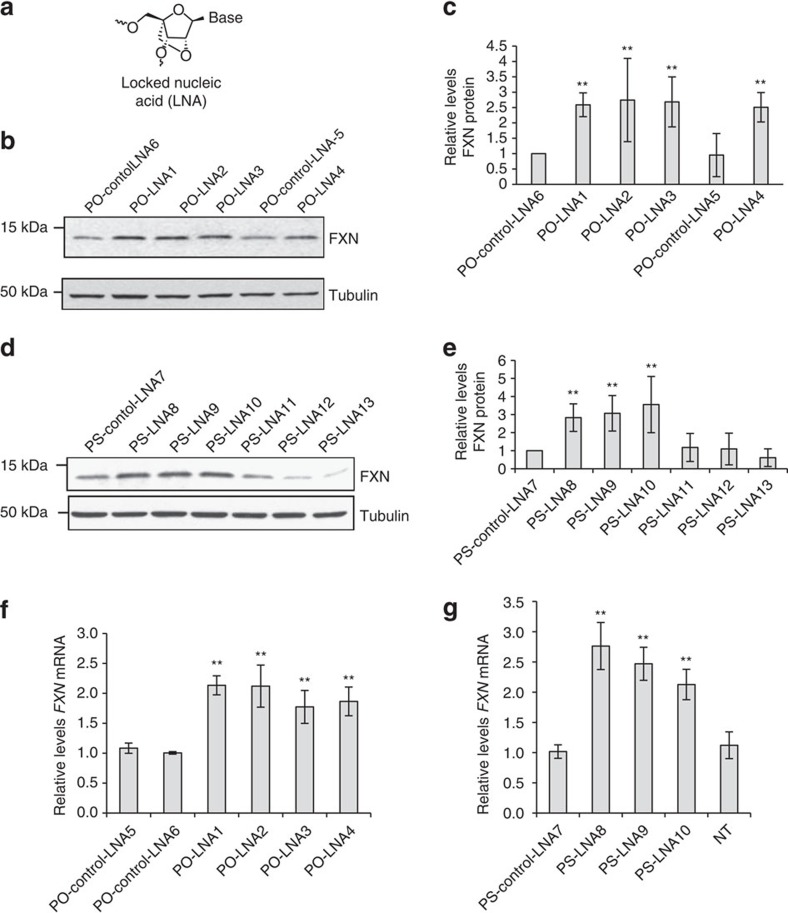
LNA-mediated activation of *FXN* expression. (**a**) Structure of LNA. (**b**) Western analysis of the effect of LNAs with phosphodiester (PO) backbone on FXN protein expression. (**c**) Quantitation of western analysis (*n*=2). (**d**) Western analysis of the effect of LNAs with phosphorothioate (PS) backbone on FXN protein expression. (**e**) Quantitation of quadruplicate western analysis. (**f**,**g**) qPCR showing effect on *FXN* mRNA expression of (**f**) PO LNAs (*n*=5) or (**g**) PS LNAs (*n*=3). PO-control-LNA5 is a negative control LNA with PO backbone that is not complementary to *FXN* RNA. PO-control-LNA6 has five mismatches relative to the repeat region within *FXN* RNA target. PS-control-LNA7 is a negative control LNA similar to PO-control-LNA5 but with PS backbone. FRDA patient fibroblast cells (GM03816) were treated with 12.5 nM duplex LNAs. Cells were collected at day 3 for RNA extraction and day 4 for protein extraction. All data are presented as mean±STDEV. NT, no treatment; **P<0.01, by Student *t*-test.

LNA is modified RNA nucleotide containing a methylene linkage between the 2′ and 4′ positions of the ribose (Fig. 4a)^31^. This constraint reduces the entropic penalty of binding and the introduction of LNA bases allows hybridization affinity to be tailored for specific applications. We initially tested LNAs possessing phosphodiester internucleotide linkages but also examined LNAs containing phosphorothioate linkages likely to possess better activities *in vivo*.

We observed that anti-GAA phosphodiester and phosphorothioate LNAs increased levels of FXN protein and RNA expression (Fig. 4b–e). By contrast, LNAs complementary to all three registers of antisense RNA (PS-LNA11,12,13) at the *FXN* locus were inert (Fig. 4d,e). Both phosphodiester and phosphorothioate anti-GAA LNAs also activated expression of *FXN* mRNA expression (Fig. 4f,g). These findings are also consistent with a mechanism involving the LNA blocking the mutant transcript and preventing interactions that upregulate gene expression. Since LNAs do not function through RNAi their activity supports that conclusion that simple binding to transcript is sufficient for activation.

## Discussion

Nucleic acid therapeutics are gaining momentum as an approach to drug development^32^. A duplex RNA for treating Transthyretin-mediated amyloidosis is now in Phase III trials^33^ and other duplex RNAs have been used successfully in primates to inhibit expression of disease genes in the central nervous system^34^. This recent clinical and preclinical progress suggests that using RNA to enhance *FXN* expression is a plausible approach for developing treatments for FRDA. LNAs are also being tested in clinical trials^32^ and provide an alternative to duplex RNAs for developing nucleic acids as therapeutic activators of *FXN* expression.

There is an urgent need to identify better approaches to treat FRDA. Our study identifies duplex RNAs and LNAs that activate *FXN* expression. These two distinct synthetic agents share at a common mechanistic feature—an ability to block the GAA repeat within FXN pre-mRNA. Activating RNAs and LNAs support involvement of the mutant transcript in repression of *FXN* expression, will be useful tools for further probing mechanism, and provide a starting point for molecular therapeutic discovery.

The molecular basis of reduced mutant FXN protein expression has been a puzzle. How can a mutation within an intron affect expression of protein derived from mature mRNA? The defect and its consequences seem at odds from the normal dogma that the direction of gene expression moves linearly from DNA to RNA to protein. In this case, an aberrant RNA seems to be affecting its own transcription.

Gromak and colleagues have provided experimental evidence for an elegant solution to this problem by demonstrating that the expanded repeat forms an R-loop with complementary DNA sequence at the FXN locus^18^,^19^. While our activating DNAs and RNAs are therapeutic leads, they also represent mechanistic probes that independently support the conclusion that formation of an R-loop is the critical step in shutting down expression of mutant FXN. R-loops induce repressive chromatin marks at many mammalian genes^35^ and blocking R-loop formation may be a general strategy for controlling gene expression.

Off-target effects are an obstacle for development of nucleic acid therapeutics. We do not completely examine the potential for off-target effects in this study except to demonstrate that we do not observe activation of interferon responsive genes (Supplementary Fig. 4) and that expression of two of the most prominent GAA-repeat containing genes is unchanged (Supplementary Fig. 5). In the future, if unacceptable off-target effects are observed, they can be mitigated by changing the position of base substitutions within the anti-GAA duplex or by introducing chemically modified bases. We note that a similar development strategy yielded RNAi-active single-stranded silencing anti-CAG RNAs that were potent allele-selective silencing agents and well tolerated in a mouse model for Huntington Disease^36^.

Duplex RNAs and LNAs recognize complementary sequences by different mechanisms. Duplex RNAs function through the RNAi pathway. LNAs do not require the assistance of specific proteins. Both duplex RNAs and LNAs, however, form Watson–Crick hybrids that block complementary sequences and prevent R-loop formation with genomic DNA. For LNAs, only compounds complementary to sense strand FXN RNA are active, further supporting the hypothesis that the expanded mutant repeat is the key target.

Our work complements the development of histone deacetylase inhibitors as a strategy for activating FXN expression. Histone deacetylase inhibitors have the advantage of being more similar to traditional small molecule drugs, simplifying some aspects of development and clinical testing and possibly reducing barriers to delivery to target tissues *in vivo*. The advantage of nucleic acids is the potential for specific recognition at the *FXN* locus. In the future it might prove interesting to test the potential for the two strategies to function synergistically.

Finally, the ability of mammalian RNAi factors to be present and functional in cell nuclei has been open to debate. Recent evidence, however, has shown that RNAi factors exist in mammalian nuclei^26^,^34^,^35^,^36^,^37^,^38^,^39^,^40^ and can be active controlling splicing^37^,^41^ or transcription^24^,^25^,^42^,^43^,^44^. Our results widen the reach of nuclear RNAi by suggesting that it can be used to target intronic RNA, interfere with R-loop formation, and release the break on transcription.

## Methods

### Tissue culture and transfection of nucleic acids

Fibroblast cells (Coriell Institute, GM03816 and GM02153) were cultured in minimum essential medium supplemented with 10% foetal bovine serum and 1% non-essential amino acid (NEAA). All cells were grown at 37 °C in 5% CO_2_. Lipofectamine RNAiMAX (Invitrogen) was used to transfect siRNAs or LNA following the manufacturer's recommended protocol in OptiMEM low-serum medium (Invitrogen). Growth media was changed to full medium after 24 h. Transfected cells were harvested 72 h after transfection for RNA extraction and quantitative PCR (qPCR) analyses, and 96 h after transfection for western blot analysis. Sequences of siRNAs and LNAs used in these studies are listed in Supplementary Tables 1 and 2 respectively.

### RNA immunoprecipitation

FRDA patient fibroblast cells (GM03816) were treated with 50 nM of siRNAs CM (control dsRNA) or siGAA. Cells were grown in 15 cm dishes harvested 3 days after transfection. Cells were harvested by treating with trypsin-EDTA solution (Invitrogen), washing with PBS and then resuspended in ice-cold hypotonic lysis buffer (10 mM Tris-HCl, pH 7.5, 10 mM NaCl, 3 mM MgCl_2_, 0.3% NP-40). After incubation on ice for 15 min and pipetting and vortexing, lysate was spun at 4 °C at 800*g* for 5 min to separate nuclei from cytoplasm. Pelleted nuclei were washed 3 × with ice-cold hypotonic lysis buffer followed by 5 min incubation on ice, pipetting and vortexing, then centrifugation at 4 °C at 100*g* for 2 min.

Nuclei were resuspended in ice-cold nuclear lysis buffer (20 mM Tris-HCl, pH 7.5, 0.15 M NaCl, 3 mM MgCl_2_, 0.3% NP-40 and 10% glycerol) supplemented with 1% Protease Inhibitor Cocktail (Roche), 5 μl ml^−1^ RNaseIn (Promega) at a final of 0.5 ml per 75 mg of original wet cell pellet weight. Nuclei were sonicated on ice at 20% power 3 × for 15 s in 4 ml volumes. After high-speed centrifugation at 4 °C to remove insoluble cell debris, the soluble fraction was kept as nuclear extract.

All extracts were aliquoted, flash-frozen in liquid nitrogen and stored at −80 °C. RIP was performed as follows^38^, using anti-Ago2 (Wako) and mouse nonspecific IgG antibodies. Briefly, 100 μl of extract is diluted to 1 ml with nuclear lysis buffer for each immunoprecipitated (IP) sample, samples were incubated with 4 μg of antibody overnight at 4 °C and then after adding Protein G Plus Protein A Agarose beads (Calbiochem) for 1–2 h at 4 °C. After washes and elution, immunoprecipitated RNA was extracted by phenol:chloroform:isoamyl alchohol (25:24:1) and dissolved in nuclease-free water, RT-qPCR was performed to measure the levels of Ago2-associated *FXN* pre-mRNA using primer pairs targeting the region of intron1-exon2 (In1Ex2), with amplification of hypoxanthine phosphoribosyltransferase (HPRT) as internal control. qPCR products were resolved on 1.5% gels (Fig. 3a). The bands visible were subsequently extracted and sequenced. Primers are listed in Supplementary Table 3.

### Western blot analysis

Cell extracts were prepared using lysis buffer supplemented with 1% Protease Inhibitor Cocktail Set I (Calbiochem) as follows: in brief, cells were collected at 300*g* for 15 min, washed once by cold PBS and then frozen overnight at −80 °C after adding lysis buffer. After the cells were thawed on ice, the samples were centrifuged at top speed for 15 min and the supernatants were kept.^30^. Protein was separated on 4–20% gradient or 15% SDS–polyacrylamide gel electrophoresis TGX pre-cast gels (BioRad). After gel electrophoresis, proteins were transferred to nitrocellulose membrane (Hybond-C Extra, GE Healthcare Life Sciences) at 100 V for 45–60 min. Membranes were blocked for 30 min at room temperature with 5% milk protein in PBS+0.05% TWEEN-20 (PBST).

Blocked membranes were incubated with the primary antibodies at 4 °C in PBST and 5% milk with rocking overnight: anti-FXN at 1:300 (Abcam, ab110328), anti-tubulin at 1:6,000–10,000 (Sigma-Aldrich, T5201). After primary antibody incubation, membranes were washed 3 times for 5 min at room temperature with PBST then incubated for 30–45 min at room temperature with HRP-conjugated anti-mouse (Jackson Laboratories, 715-035-150) in PBST+5% milk. Membranes were washed again 3x for 15 min in PBST at room temperature, then protein bands visualized using SuperSignal West Pico Chemiluminescent Substrate (Thermo Scientific). For quantification of protein levels from Western blots of cellular fractions, films were scanned and bands quantified using ImageJ. An uncropped scan for one of FXN blots with size marker is presented in Supplementary Fig. 9.

### Quantitative PCR

Identical volumes of RNA (representing approximately the same number of cells and ranging from 1 to 2 μg of RNA) were treated with 2 units of DNase I (Worthington) in DNase I buffer (10 mM Tris-HCl, pH 7.0, 10 mM NaCl, 2 mM MgCl_2_ and 0.5 mM CaCl_2_) for 15 min at room temperature to degrade any genomic DNA contamination. Afterwards, DNase I heat-inactivated at 75 °C for 10 min. Treated RNAs were reverse-transcribed using the High Capacity cDNA Reverse Transcription Kit (Applied Biosystems). qPCR was performed using SYBR Supermix (Biorad) with ∼50 ng of cDNA as template. Data were normalized relative to measured HPRT levels.

### Chromatin immunoprecipitation

FRDA GM03816 cells were transfected with siGAA or CM and cultured as described above for RIP. Three days after transfection, cells were crosslinked with 1% formaldehyde and harvested by scraping. Cell nuclei were isolated by washing twice with hypotonic lysis buffer (5 ml X2; 10 mM Tris-HCl (pH 7.5), 10 mM NaCl, 3 mM MgCl_2_ and 0.5% NP-40). Nuclei were lysed in nuclear lysis buffer (1 ml; 50 mM Tris-HCl (pH 8.1), 10 mM EDTA, 1% SDS, 1X protease inhibitors cocktail (Roche) and then sonicated (3 pulses, 20% power, 20 s). Each sample was centrifuged at 18,928*g* for 10 min at 4 °C and the supernatant was kept as nuclear lysate. The nuclear lysate (40 μl) was incubated overnight with antibodies in immunoprecipitation buffer (1 ml; 16.7 mM Tris-HCl (pH 8.1), 167 mM NaCl, 1.2 mM EDTA, 1.1% Triton X-100, 0.01% SDS and 1X protease inhibitor cocktail). The antibodies used for ChIP are as follows: anti-RNAP2 (2 μg; Millipore, 05-623), anti-H3K4me3 (3 μg; Abcam, ab8580), anti-H3K9me2 (3 μl; Millipore, 17-648), anti-H3K9me3 (3 μg; Millipore, 17–625), anti-H3K27me3 (3 μg; Millipore, 17–449), anti-H3K9Ac (3 μg; Millipore, 17–658), anti-H4Ac (K5, 8, 12, 16; 3 μg; Millipore, 06-598), normal rabbit IgG (3 μg; Millipore, 12–370) and normal mouse IgG (2 or 3 μg; Millipore, 12–371). After that, each sample was incubated with 50 μl of Protein G plus Protein A Agarose Beads (Calbiochem) at 4 °C for 3 h. The beads were washed with low-salt buffer (20 mM Tris-HCl (pH 8.1), 150 mM NaCl, 2 mM EDTA, 1% Triton X-100 and 0.1% SDS), high-salt buffer (see low salt but with 500 mM NaCl), LiCl solution (10 mM Tris-HCl (pH 8.1), 1 mM EDTA, 1% deoxychorate, 1% NP-40, 0.25 M LiCl) and TE buffer (pH 8.0). Protein was eluted twice with 250 μl of elution buffer (0.1 M NaHCO3, 1% SDS) for 5 min at room temperature. Reverse crosslinking was performed by adding NaCl to 200 mM and heating at 65 °C for 2 h 30 min. Each sample was treated with proteinase K (20 μg) at 42 °C for 50 min, followed by phenol extraction using an equal volume of phenol/chloroform/isoamyl alcohol. DNA in the aqueous layer was precipitated using 1/10 volume 3 M sodium acetate (pH 5.5), 2.2 volumes 100% ethanol, and glycogen (30 μg). The pellets were dissolved in 100 μl of nuclease-free water and then used as templates for qPCR. The sequences of the qPCR primers are presented in Supplementary Table 3.

### HDAC inhibitor and mRNA stability assay

Histone deacetylase inhibitor BML210 (Abcam; 5 μM) was applied to FRDA fibroblast cells (GM03816) by following the procedure described in Herman *et al.*^27^. In brief, cells were incubated with BML210 (dissolved in DMSO) or DMSO only for 3 days (for RNA) or 4 days (for protein). Cells were collected for qPCR and Western blot analyses as mentioned above. For mRNA stability assay, cells were transfected with siGAA or CM as above, and 5 μg ml^−1^ actinomycin D (Sigma) was added with fresh media 3 day after transfection, and cells were collected at the indicated time points for RNA extraction and qPCR analysis.

### DIP analysis

FRDA patient fibroblast cells (GM03816) were treated with 50 nM siRNAs CM and siGAA as above. Cells were collected 2 day afternoon transfection. Cell lysis and nuclei collection were as described above in RIP section. The purified nuclei were lysed and further processed for IP as described^18^, except the proteinase K (Invitrogen) treatment were carried out at 42 °C. Briefly, nuclei were incubated on ice in nuclei lysis buffer (50 mM TRIS pH 8.0, 5 mM EDTA and 1% SDS), and proteins and cell debris were removed by centrifugation after incubation with proteinase K. Following isopropanol precipitation and 70% ethonal wash, DNA was resuspended in IP dilution buffer (16.7 mM TRIS pH 8.0, 1.2 mM EDTA, 167 mM NaCl, 0.01% SDS and 1.1% Triton X-100) and sonicated to the size ∼500 bp. DIP was performed by DNA–RNA specific antibody S9.6 (Kerafast) following published methods^18^. Briefly, 4 μl of S9.6 antibody was added to ∼25–40 μg DNA pre-cleared with Protein G Plus Protein A Agarose beads (Calbiochem, 1 h at 4 °C) incubated overnight at 4 °C. After further incubated with 60 μl Protein G Plus Protein A Agarose beads (Calbiochem, 2 h at 4 °C), followed by washes and elution, the extracted DNA was dissolved in nuclease-free water. qPCR analyses for the immunoprecipitated and control DNAs were performed and analysed as described^18^,^45^. Briefly, the DIP signal of immunoprecipitated RNA/DNA hybrid (R-Loop) was quantified by first subtracting the background signal, which was obtained from the control without using antibody, then compared with the input. FXNin1UP is the region adjacent to the GAA-repeat region of *FXN* intron 1. *ZNF554* is a non-R-loop-forming genomic locus (HGNC:26629) serving as a negative control; while *MYADM* is a strong R-loop-forming locus (HGNC:7544) as a positive control. The primer pairs for qPCR are: FXNin1UP (F: 5′-GAAACCCAAAGAATGGCTGTG-3′; R: 5′-TTCCCTCCTCGTGAAACACC-3′); *ZNF554* (F: 5′-CGGGGAAAAGCCCTATAAAT-3′; R: 5′-TCCACATTCACTGCATTCGT-3′); *MYADM* (F: 5′-CGTAGGTGCCCTAGTTGGAG-3′; R: 5′-TCCATTCTCATTCCCAAACC-3′).

## Additional information

**How to cite this article:** Li, L. *et al.* Activating frataxin expression by repeat-targeted nucleic acids. *Nat. Commun.* 7:10606 doi: 10.1038/ncomms10606 (2016).

## Supplementary Material

Supplementary Information Supplementary Figures 1-9 and Supplementary Tables 1-3
