# Supplementary material for: Activating frataxin expression by repeat-targeted nucleic acids
Source: Nat Commun. 2016 Feb 4;7:10606. doi: 10.1038/ncomms10606 (PMC4742999; doi:10.1038/ncomms10606)
Supplement: Supplementary Information — Supplementary Figures 1-9 and Supplementary Tables 1-3 [file ncomms10606-s1.pdf]

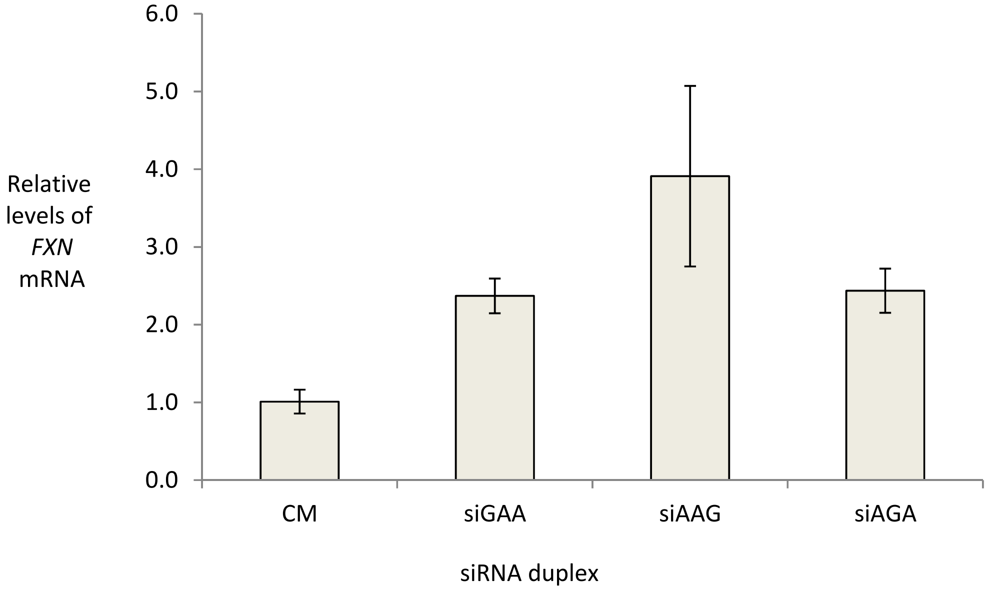

Supplementary Figure 1A. Effects of anti-GAA duplex RNAs on *FXN* mRNA expression. FRDA patient fibroblast cells (GM03816) were treated with 50 nM of siRNAs. siGAA, siAAG and siAGA are duplex RNAs complementary to the expanded repeat in three different registers. CM is a negative control RNAs that is not complementary to *FXN* RNA. Data are presented as Mean ± STDEVP, n=3. **: P< 0.01

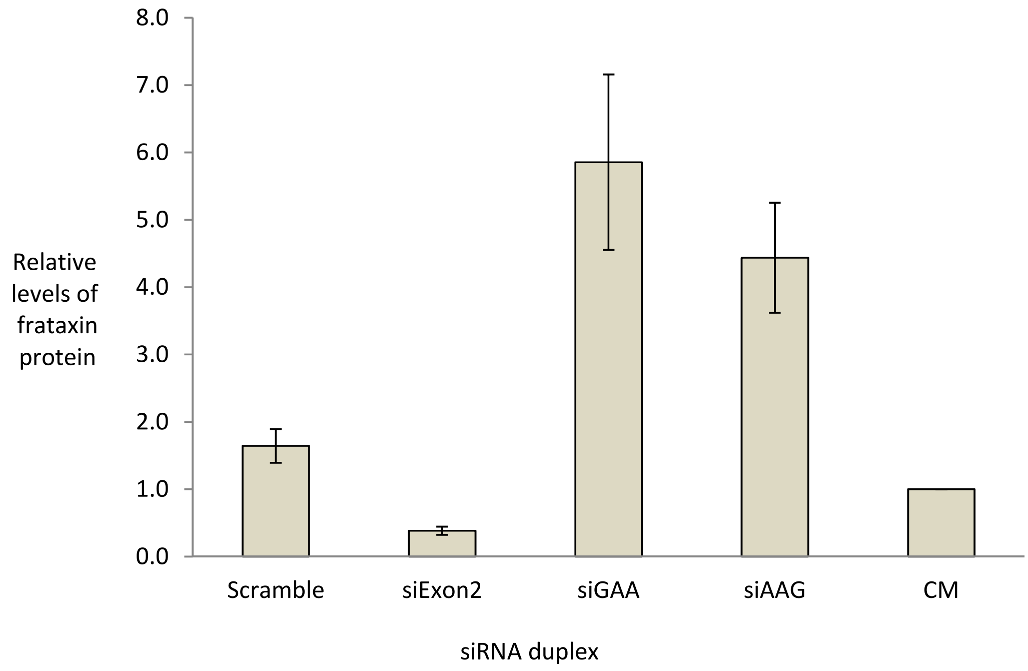

Supplementary Figure 1B. Effects of anti-GAA duplex RNAs on FXN protein expression. FRDA patient fibroblast cells (GM03816) were treated with 50 nM of siRNAs. siGAA, siAAG are duplex RNAs complementary to the expanded repeat in three different registers. CM is a negative control RNAs that is not complementary to *FXN* RNA. Data are presented as Mean ± SE, n=7. **: $P < 0.01$

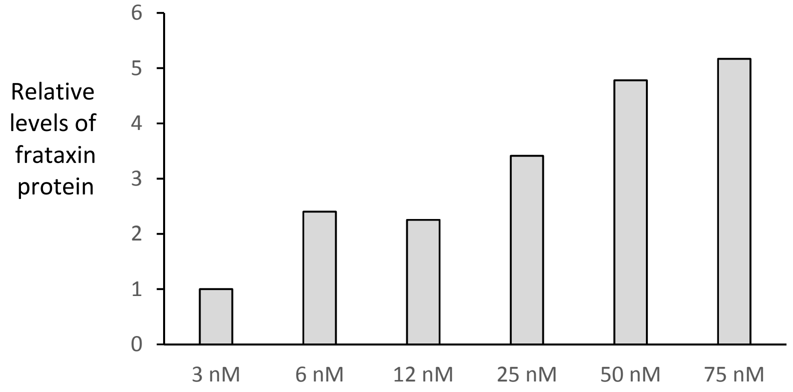

Supplementary Figure 1C. Dose curve for siGAA on FXN protein expression corresponding to Figure 1F. FRDA patient fibroblast cells (GM03816) were treated with different concentration of siRNAs.

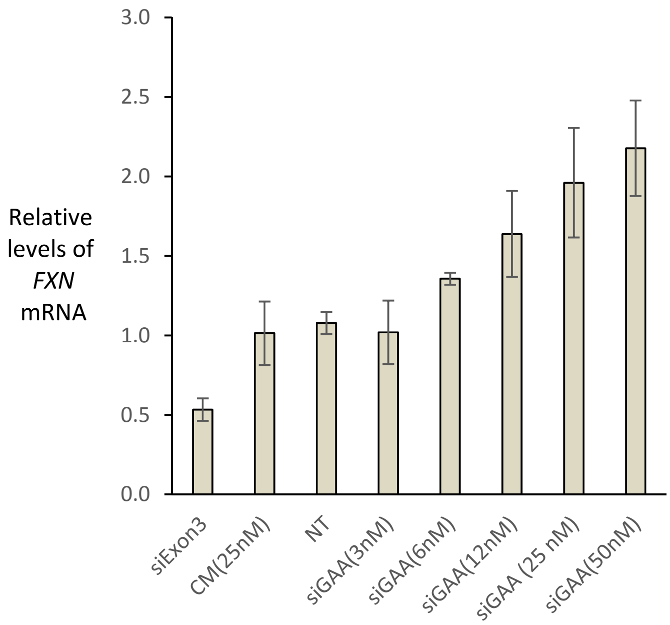

Supplementary Figure 1D. Dose curve for siGAA effects on *FXN* mRNA expression. FRDA patient fibroblast cells (GM03816) were treated with different concentration of siRNAs. NT: no treatment. Data are presented as Mean ± SD, n=2.

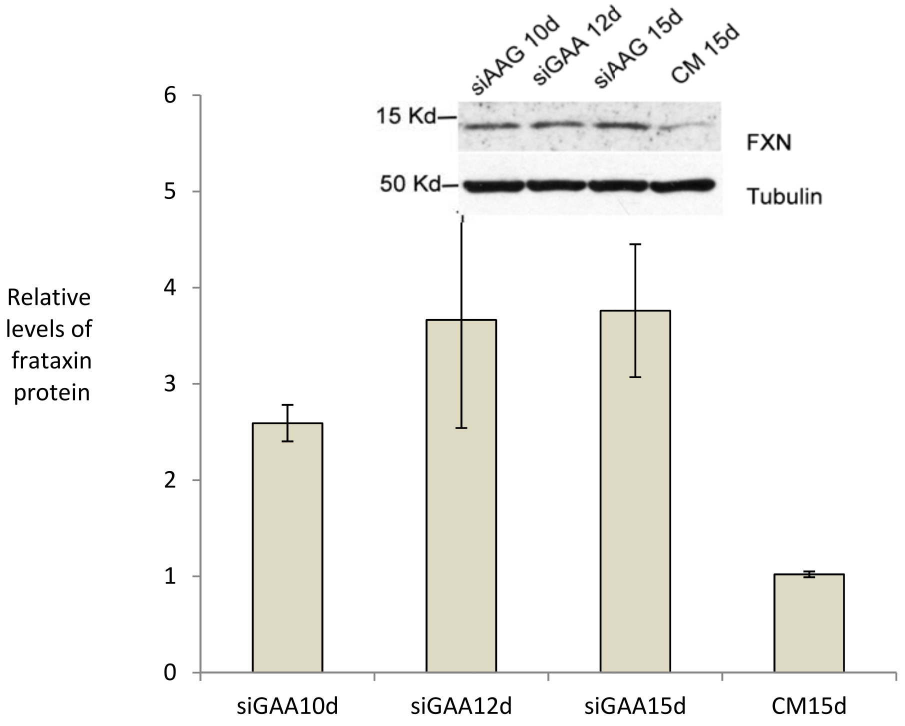

Supplementary Figure 2. Effects of anti-GAA duplex RNAs on FXN protein expression over time. FRDA patient fibroblast cells (GM03816) were treated with 50 nM of siRNAs. Data are presented as Mean ± SD, n=2.

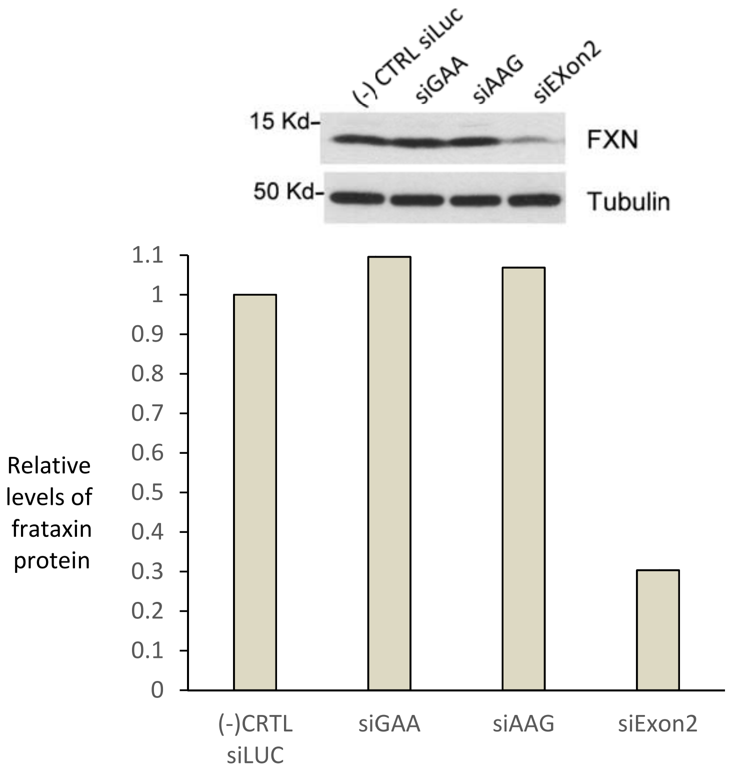

Supplementary Figure 3. Anti-GAA duplex RNAs do not influence FXN protein expression in wild-type cells. siLuc is a negative control duplex siRNA that is not complementary to *FXN* RNA and has no target in the genome. For the western analysis, protein sample of each treatment was collected from 3 individual tranfections 4 days after tranfection of wild-type fibroblast cells (GM02153) with 25 nM siRNAs.

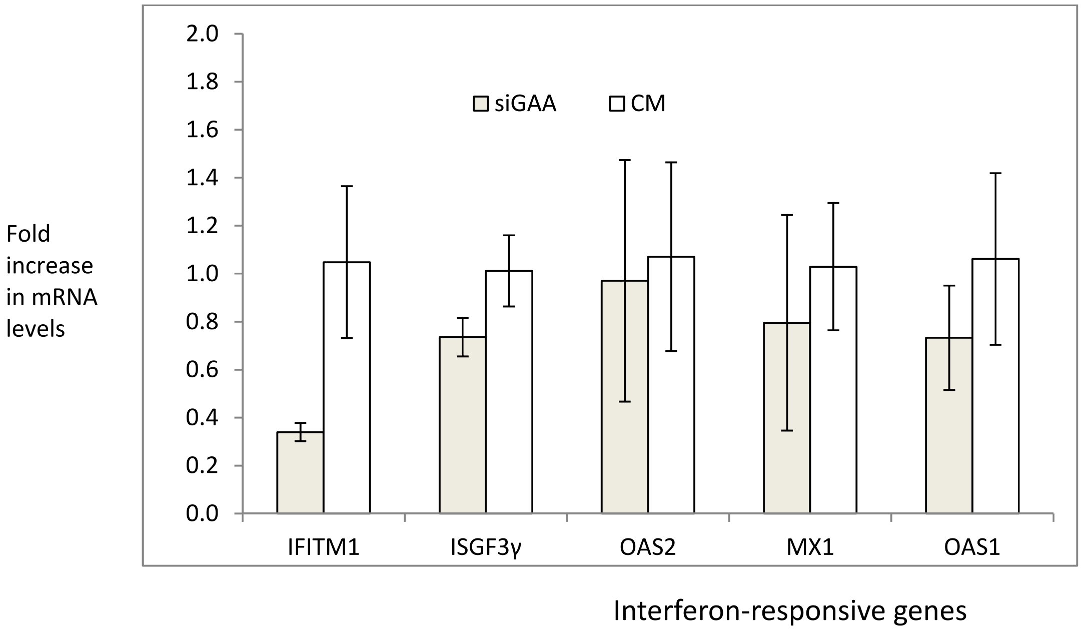

Supplementary Figure 4. siGAA does not induce expression of interferon responsive genes. qPCR data showing no significant impact of siGAA compared with CM control on the mRNA expression of interferon responsive genes. FRDA patient fibroblast cells (GM03816) were treated with 25nM of siRNAs, and were collected for qPCR 3 days after transfection. Data are presented as Mean ± SD, n=2. GAPDH was used as internal control for the interferon responsive genes.

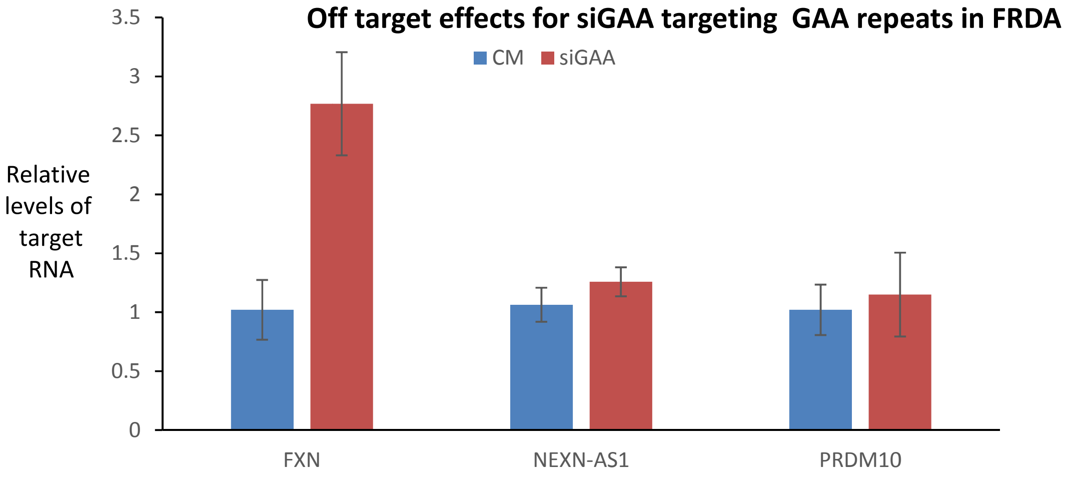

siGAA off-target candidates		GAA repeat (3nt)#	Possible binding site # for siRNA (19 nt)	Off target effect tested by qPCR ?
Homo sapiens NEXN antisense RNA 1 (NEXN-AS1), long non-coding RNA		32	5	No effect
PREDICTED: Homo sapiens PR domain containing 10 (PRDM10), mRNA	transcript variant X5	10	1	No effect
	transcript variant X3	10	1	
	transcript variant X2	10	1	
	transcript variant X1	10	1	
	transcript variant X4	9	1	
PREDICTED: Homo sapiens transglutaminase 5 (TGM5), transcript variant X2, mRNA		7	1	Expression level too low to be detectable for all samples

Supplementary Figure 5. Effects of anti-GAA duplex RNA on target RNA expression. FRDA patient fibroblast cells (GM03816) were treated with 50 nM of siRNAs in 15 cm plates. siGAA is duplex RNA complementary to the expanded repeat. CM is a negative control RNAs that is not complementary to *FXN* RNA. Data are presented as Mean ± SD, n=3.

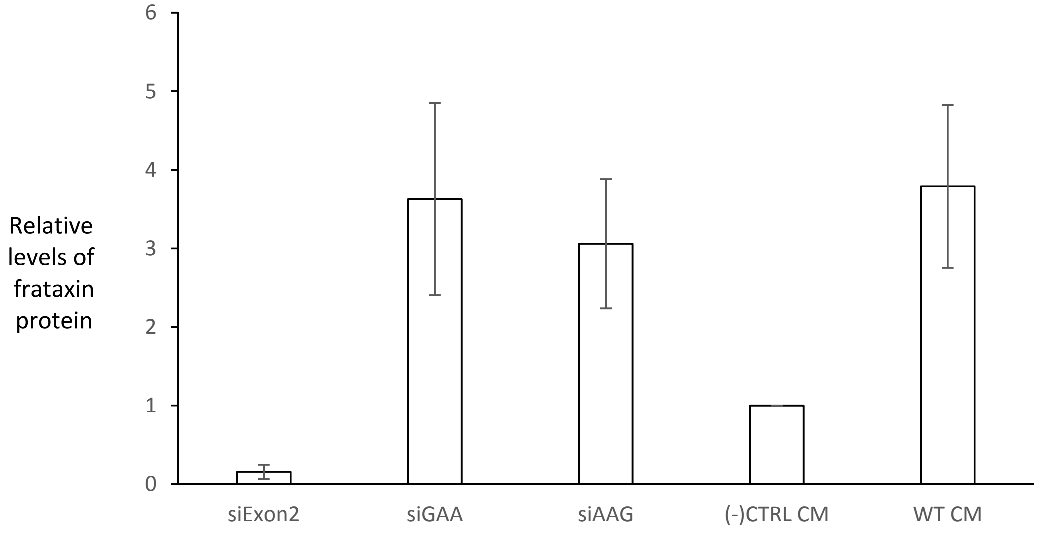

Supplementary Figure 6. Comparison of RNA-mediated activation of FXN expression in patient cells to FXN expression in normal cells. FRDA patient fibroblast cells (GM03816) and wild-type fibroblast cells (GM02513)were treated with 50 nM of siRNAs. Data are presented as Mean ± SD, n=3.

Ago2 RIPed sequence

TATGCATTAATGGGTTATAATTCACTGAAAAATAGTAACGTACTTCTTAACTTTGGCTTTCAGA
GTTCGAACCAACGTGGCCTCAACCAGATTTGGAATGTCAAAAAGCAGAGTGTCTATTTGATGAA
TTTGAGGAAATCTGGAACTTTGGGC

Green: *FXN* intron1 region; Red: *FXN* Exon2 region; **Bold** and ***italic***: sequenced region

Sequenced Ago2IP sequence

>Ago2IP sequence

```
NNNNNNNNNNNANNNNNNNNNTATGCATTAATGGGTTATAATTCACTGAAAAATAGTAACGTACTTCTTAACTTTGGCTT
TCAGAGTTCGAACCAACGTGGCCTCAACCAGATTTGGAATGTCAAAAAGCAGAGTGTCTATTTGATGAATTTGAGGAAAT
CTGGAACTTTGGGCNNNGANNTTCTNNNGTCCGGGCGNCCNNNTCTCCNCCCNGNCTCNCTGCATNCNTGGAGACCCAGT
CNCCNTCCCCGTNCTGNNNCNGNNGCATNANGANTNGGCNCCNNNTNNTTTTTNAGCCGGANNCNGNNTNTCACCNGNNC
NNGCCNGCANNNCNTTGCNTCTTNTNTGTNCAGCNGTNN
Query  22         TATGCATTAATGGGTTATAATTCACTGAAAAATAGTAACGTACTTCTTAACTTTGGCTTT  81
                  ||||||||||||||||||||||||||||||||||||||||||||||||||||||||||||
Sbjct  69046322   TATGCATTAATGGGTTATAATTCACTGAAAAATAGTAACGTACTTCTTAACTTTGGCTTT  69046381
Query  82         CAGAGTTCGAACCAACGTGGCCTCAACCAGATTTGGAATGTCAAAAAGCAGAGTGTCTAT  141
                  ||||||||||||||||||||||||||||||||||||||||||||||||||||||||||||
Sbjct  69046382   CAGAGTTCGAACCAACGTGGCCTCAACCAGATTTGGAATGTCAAAAAGCAGAGTGTCTAT  69046441
Query  142        TTGATGAATTTGAGGAAATCTGGAACTTTGGGC  174      Sequenced Ago2 IP sequence
                  |||||||||||||||||||||||||||||||||
Sbjct  69046442   TTGATGAATTTGAGGAAATCTGGAACTTTGGGC  69046474 FXN gene locus
```

Supplementary Figure 7. *FXN* product obtained through Ago2-RIP. The Ago2-associated *FXN* pre-mRNA products were obtained as present in Figure 3(e), and extracted and sequenced. Green color indicates nucleotides belonging to *FXN* intron1 region, and red color indicates nucleotides belonging to *FXN* Exon2 region.

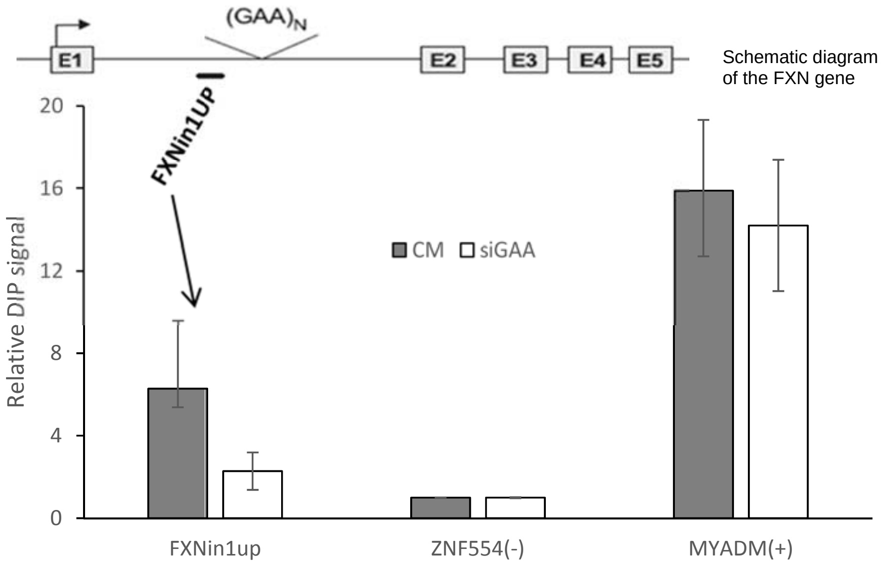

Supplementary Figure 8 Effects of anti-GAA duplex RNAs on R-Loop formation at intron 1 repeat region. FRDA patient fibroblast cells (GM03816) were treated with siRNAs CM and siGAA. siGAA is duplex RNA complementary to the expanded repeat in three different registers. CM is a negative control RNAs that is not complementary to *FXN* RNA. DNA IP (DIP) was performed by DNA-RNA specific antibody S9.6 (Kerafast). FXNin1UP is the region adjacent to the GAA repeat region of *FXN* intron 1. ZNF554 is a a non-R-loop-forming genomic locus(HGNC:26629) serving as a negative control; while MYADM is a strong R loop-forming locus (HGNC:7544) as a positive control (Loomis EW, Sanz LA, Che´din F, Hagerman PJ (2014) Transcription-Associated R-Loop Formation across the Human FMR1 CGG-Repeat Region. PLoSGenet 10(4): e1004294. doi:10.1371/journal.pgen.1004294). Data are presented as Mean ± SE, n=5

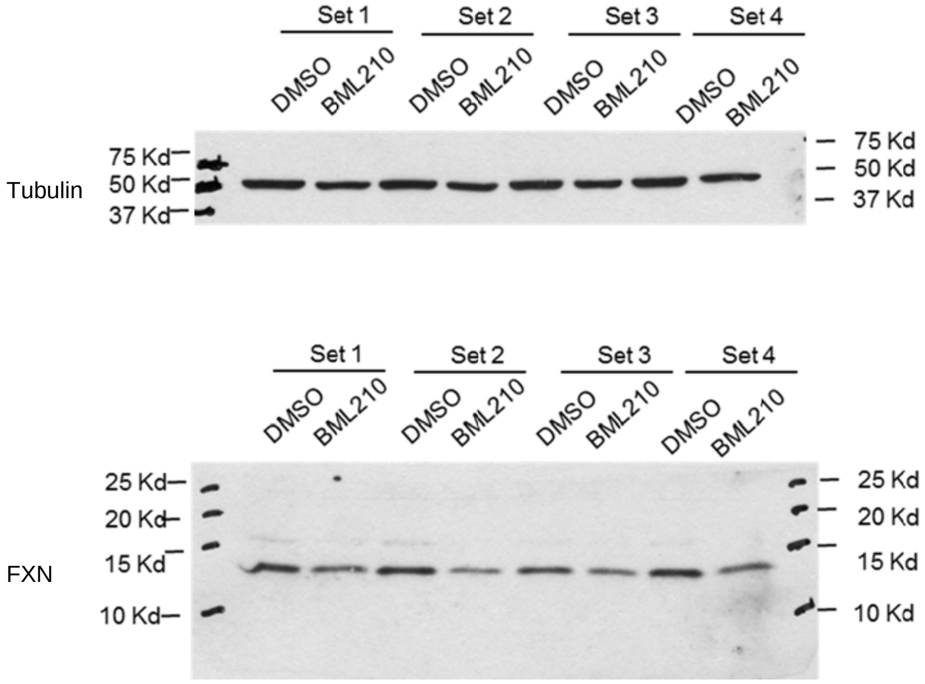

Supplementary Figure 9 . Uncropped images for effect of a histone deacetylase inhibitor on FXN expression. Effect of HDAC inhibitor BML210 (5 µM) treatment on expression of protein expression in FRDA patient fibroblast cells (GM03816).

Supplementary Table 1. siRNAs targeting the FXN locus.

RNA duplex	Strand	Sequence	Tm for duplex, °C
siGAA	ss	GAAGAAGAAGAAGAAGAAGdTdT	65.64
	as	CUUCUUCUUCUUCUUCUUCdTdT	
siAAG	ss	AAGAAGAAGAAGAAGAAGAdTdT	64.67
	as	UCUUCUUCUUCUUCUUCUUdTdT	
siAGA	ss	AGAAGAAGAAGAAGAAGAAdTdT	64.3
	as	UUCUUCUUCUUCUUCUUCUdTdT	
siExon2	ss	GAGUGUCUAUUUGAUGAAUdTdT	64.53
	as	AUUCAUCAAAUAGACACUCdTdT	
scramble	ss	GAGAAAGAGAAAAGGAAAGdTdT	66.13
	as	CUUUCCUUUUCUCUUUCUCdTdT	
siAAG5mm	ss	AA*C*AA*T*AA*T*A*C*G*C*AGAAGAdTdT	63.24
	as	UCUUCU*G*C*G*U*A*UU*A*UU*G*UUdTdT	
siGAA9,10mm	ss	GAAGAAGA *UC* AAGAAGAAGdTdT	54.4
	as	CUUCUUCUU *AG* UCUUCUUCdTdT	
siExon3	ss	GACCACCUAUGAAAGACUA dTdT	73.65
	as	UAGUCUUUCAUAGGUGGUC dTdT	

The individual strand is shown from 5' to 3'. Mismatched bases are underlined, in red, and in italics. Both strands of the duplexes containing two TT overhang. siRNAs were tested in FRDA patient fibroblasts GM03816 unless otherwise noted.

Supplementary Table 2 LNAs used in this study:

LNA	Sequence (5’-3’)<br><br>LNA (Bold,underlined), DNA (capital)
LNA with PO backbone	
PO-LNA1	**C**TT**C**TT**C**TT**C**TT**C**TT**C**TT**C**
PO-LNA2	**T**C**TT**C**TT**C**TT**C**TT**C**TT**C**TT**
PO-LNA 3	TT**C**TT**C**TT**C**TT**C**TT**C**TT**C**T
PO-LNA4	T**C**TT**C**TT**C**TT**C**TT**C**TT**C**TT
PO-control-LNA5	GCTATACCAGCGTCGTCAT
PO-control-LNA6	**T**C**TT**C**TG**C**GT**A**TT**A**TT**G**TT**
LNA with PS backbone	
PS-control-LNA7	GCTATACCAGCGTCGTCAT
PS-LNA8	**C**TT**C**TT**C**TT**C**TT**C**TT**C**TT**C**
PS-LNA9	**T**C**TT**C**TT**C**TT**C**TT**C**TT**C**TT**
PS-LNA10	TT**C**TT**C**TT**C**TT**C**TT**C**TT**C**T
PS-LNA11	G**AA**G**AA**G**AA**G**AA**G**AA**G**AA**G
PS-LNA12	AA**G**AA**G**AA**G**AA**G**AA**G**AA**G**A
PS-LNA13	**A**G**AA**G**AA**G**AA**G**AA**G**AA**G**AA**

Supplementary Table 3 qPCR primers for *FXN* locus

Name	Sequence	Reference
FXNF	aagccatacacgtttgaggacta	35
FXNR	ttggcgtctgcttgttgatca	
PromoterF	ccccacatacccaactgctg	36
PromoterR	gcccgccgcttctaaaattc	
Ex1UpF	aagcaggctctccatttttg	36
Ex1UpR	ccgcaggcactcttctgt	
In1_UpF	atggctgtggggatgaggaagat	36
In1_UpR	tgcccagacggttccctcctc	
In1_DownF	gcatctctggaaaaataggcaagtgt	36
In1_DownR	caggggtggaagcccaatacg	
In1Ex2F	agcactcggttacaggcact	36
In1Ex2R	gcccaaagttccagatttcc	
HsHprt1F	agttctgtggccatctgcttagtag	35
HsHprt1R	aaacaacaatccgcccaaagg	

35. ChapdelaineP., Coulombe, Z.,, Chikh, A.,, Gerard, C., & Tremblay, J. P. A Potential New Therapeutic Approach for Friedreich Ataxia: Induction of Frataxin Expression With TALE Proteins. *Mol Ther Nucleic Acids,* **2**, e119(2013).
36. Kim, E., Napierala, M., & Dent, S. Y. R. Hyperexpansion of GAA repeats affects post-initiation steps of FXN transcription in Friedreich's ataxia. *Nucleic Acids Research,* **39**, 8366-8377(2011) .
